# A Comparison of Micro-CT and Dental CT in Assessing Cortical Bone Morphology and Trabecular Bone Microarchitecture

**DOI:** 10.1371/journal.pone.0107545

**Published:** 2014-09-16

**Authors:** Jui-Ting Hsu, Ying-Ju Chen, Jung-Ting Ho, Heng-Li Huang, Shun-Ping Wang, Fu-Chou Cheng, Jay Wu, Ming-Tzu Tsai

**Affiliations:** 1 School of Dentistry, College of Medicine, China Medical University, Taichung, Taiwan; 2 Stem Cell Medical Research Center, Department of Medical Research, Taichung Veterans General Hospital, Taichung, Taiwan; 3 Department of Orthopaedics, Taichung Veterans General Hospital, Taichung, Taiwan; 4 Department of Biomedical Imaging and Radiological Science, China Medical University, Taichung, Taiwan; 5 Department of Biomedical Engineering, Hungkuang University, Taichung, Taiwan; Université Jean Monnet, France

## Abstract

**Objective:**

The objective of this study was to evaluate the relationship between the trabecular bone microarchitecture and cortical bone morphology by using micro-computed tomography (micro-CT) and dental cone-beam computed tomography (dental CT).

**Materials and Methods:**

Sixteen femurs and eight fifth lumbar vertebrae were collected from eight male Sprague Dawley rats. Four trabecular bone microarchitecture parameters related to the fifth lumbar vertebral body (percent bone volume [BV/TV], trabecular thickness [TbTh], trabecular separation [TbSp], and trabecular number [TbN]) were calculated using micro-CT. In addition, the volumetric cancellous bone grayscale value (vCanGrayscale) of the fifth lumbar vertebral body was measured using dental CT. Furthermore, four cortical bone morphology parameters of the femoral diaphysis (total cross-sectional area [TtAr], cortical area [CtAr], cortical bone area fraction [CtAr/TtAr], and cortical thickness [CtTh]) were calculated using both micro-CT and dental CT. Pearson analysis was conducted to calculate the correlation coefficients (*r*) of the micro-CT and dental CT measurements. Paired-sample *t* tests were used to compare the differences between the measurements of the four cortical bone morphology parameters obtained using micro-CT and dental CT.

**Results:**

High correlations between the vCanGrayscale measured using dental CT and the trabecular bone microarchitecture parameters (BV/TV [*r* = 0.84] and TbTh [*r* = 0.84]) measured using micro-CT were observed. The absolute value of the four cortical bone morphology parameters may be different between the dental CT and micro-CT approaches. However, high correlations (*r* ranged from 0.71 to 0.90) among these four cortical bone morphology parameters measured using the two approaches were obtained.

**Conclusion:**

We observed high correlations between the vCanGrayscale measured using dental CT and the trabecular bone microarchitecture parameters (BV/TV and TbTh) measured using micro-CT, in addition to high correlations between the cortical bone morphology measured using micro-CT and dental CT. Further experiments are necessary to validate the use of dental CT on human bone.

## Introduction

Human bones are generally classified into cortical bone (synonymous with compact bone) and cancellous bone (synonymous with trabecular bone or spongy bone). The two types are classified based on porosity and the unit microstructure. Cortical bone is much denser than cancellous bone with a porosity ranging between 5% and 30% [1]. Cortical bone is primarily located in the shaft of long bones and forms the outer shell around cancellous bone (vertebrae or pelvis). Cancellous bone is considerably more porous than cortical bone with a porosity ranging between 30% and 90% [1]. It is located at the end of long bones and vertebrae, and in flat bones such as the pelvis.

Bone quality and quantity are affected by numerous factors, such as age, hormones, arthritis, and exercise. Clinically, orthopedic physicians commonly use dual energy X-ray absorptiometry (DXA) to measure the bone mineral density (BMD) of the femoral neck or spine for determining patients' bone strength [2]. Bone strength is affected by both geometric parameters and densitometric parameters. However, DXA provides only areal BMD information [3], [4], and does not include geometric parameters, such as size and shape. Although quantitative computed tomography (QCT) can provide both geometric and densitometric parameters [2], [5], the clinical application of this technique is not extensive because of the cost and high radiation dosage.

In the past two decades, microcomputed tomography (micro-CT) has been extensively used in the study of bone tissue [6]–[14]. Except for the densitometric parameters (volumetric BMD), the geometric parameters of bone can be precisely detected using micro-CT; for example, total cross-sectional area (TtAr), cortical area (CtAr), cortical bone area fraction (CtAr/TtAr), and cortical thickness (CtTh) can be detected [15]. Furthermore, micro-CT can provide detailed information on the trabecular bone, such as percent bone volume (BV/TV), bone specific surface (BS/BV), trabecular thickness (TbTh), trabecular bone separation (TbSp), and mean trabecular bone number (TbN) [15]. Therefore, micro-CT can be considered the gold standard for evaluating trabecular bone structure. However, micro-CT cannot be applied on humans because of the small scanning range [16].

Recently, dental cone-beam computed tomography (dental CT) has been widely used to evaluate alveolar bone density prior to dental implant placement [17]–[23]. Nomura et al. [24] indicated that dental CT could be used to evaluate bone mineral content based on the voxel values. In addition, numerous researchers have used the grayscale of dental CT to represent bone density (bone density in grayscale value), which is also called radiographic bone density [20]–[23]. However, most of these researchers have used dental CT in dental-related research or clinical trials. In our previous study [16], we indicated that dental CT is superior to DXA for predicting cortical bone fracture loads in rat femurs and tibias. Nevertheless, the relation between the bone density in grayscale measured using dental CT and trabecular bone microarchitectures is still unclear. Therefore, the purpose of this study was to evaluate the relationship between cortical bone morphology and trabecular bone microarchitecture by using micro-CT and dental CT.

## Materials and Methods

### Specimen preparation

Sixteen femurs and eight fifth lumbar vertebrae were collected from eight 4-month-old healthy male Sprague Dawley rats. All rats were killed by carbon dioxide asphyxiation, the entirety of the femurs and fifth lumbar vertebrae were harvested from every rat within 20 min. The bone specimens were wrapped with gauze soaked in saline and stored in a −20°C freezer. The study procedures were conducted in strict accordance with the recommendations provided in the Guide for the Care and Use of Laboratory Animals of the National Institutes of Health. We obtained animal research ethics approval from the Research Ethics Committee of the Taichung Veterans General Hospital (Permit Number: La-1021069).

### Micro-CT measurement

**Figure 1:**
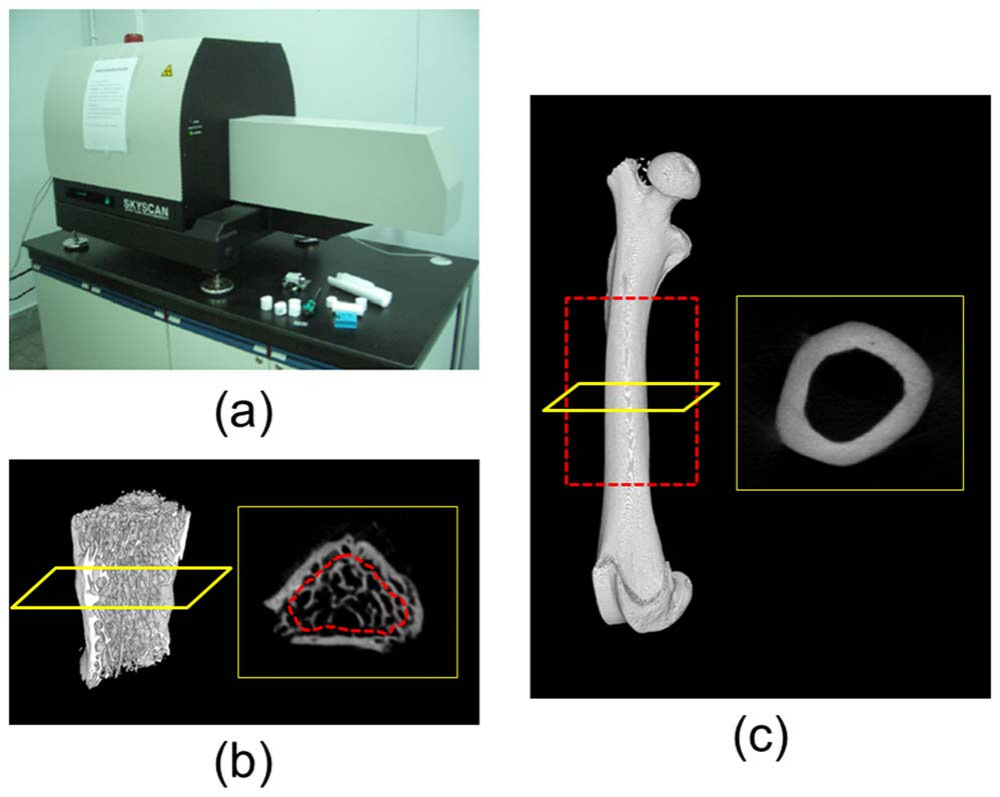
Micro-CT machine, images and 3D renderings. (a) Micro-CT machine (Skyscan 1076), (b) hemi 5th lumbar vertebral body (excluded the posterior element) in left, the section of the micro-CT image in right (the region of interest for trabecular bone calculated also shown in dotted red line), and (c) intact femur (the region of interest for cortical bone calculated also shown in dotted red line) in left, the section of the micro-CT image in right.

The micro-CT images of each femur and fifth lumbar vertebrae were obtained using a Skyscan 1076 micro-CT device (Skyscan, Aartselaar, Belgium) (Fig. 1a). The scanning parameters were set at 49 kV, 200 µA, 500 ms, and a voxel resolution of 18.27 µm. The micro-CT images were imported into CTAn software (Skyscan) to measure the four parameters of trabecular bone microarchitecture: BV/TV, TbTh, TbSp, and TbN of the cancellous bone in the fifth lumbar vertebral body (Fig. 1b). Furthermore, four parameters of cortical bone morphology, TtAr, CtAr, CtAr/TtAr, and CtTh of the femoral diaphysis, were calculated (Fig. 1c) using ImageJ (Rasband, W.S., ImageJ, U.S. National Institutes of Health, Bethesda, MD, USA). The parameters of trabecular bone microarchitecture and cortical bone morphology measured in this study are listed in Table 1.

**Table 1:** Definition and description of parameters for trabecular bone microarchitecture and cortical bone morphology.

Bone type	Abbreviation	Description	Unit
Trabecular bone (5^th^ lumbar vertebral body)	BV/TV	Bone volume fraction: Ratio of the segmented bone volume to the total volume of the region of interest	%
	TbTh	Trabecular thickness: Mean thickness of trabeculae	mm
	TbSp	Trabecular separation: Mean distance between trabeculae	mm
	TbN	Trabecular number: Measure of the average number of trabeculae per unit length	1/mm
Cortical bone (femoral diaphysis)	TtAr	Total cross-sectional area inside the periosteal envelope	mm^2^
	CtAr	Cortical bone area	mm^2^
	CtAr/TtAr	Cortical area fraction	%
	CtTh	Average cortical thickness	mm

### Dental CT measurement

**Figure 2:**
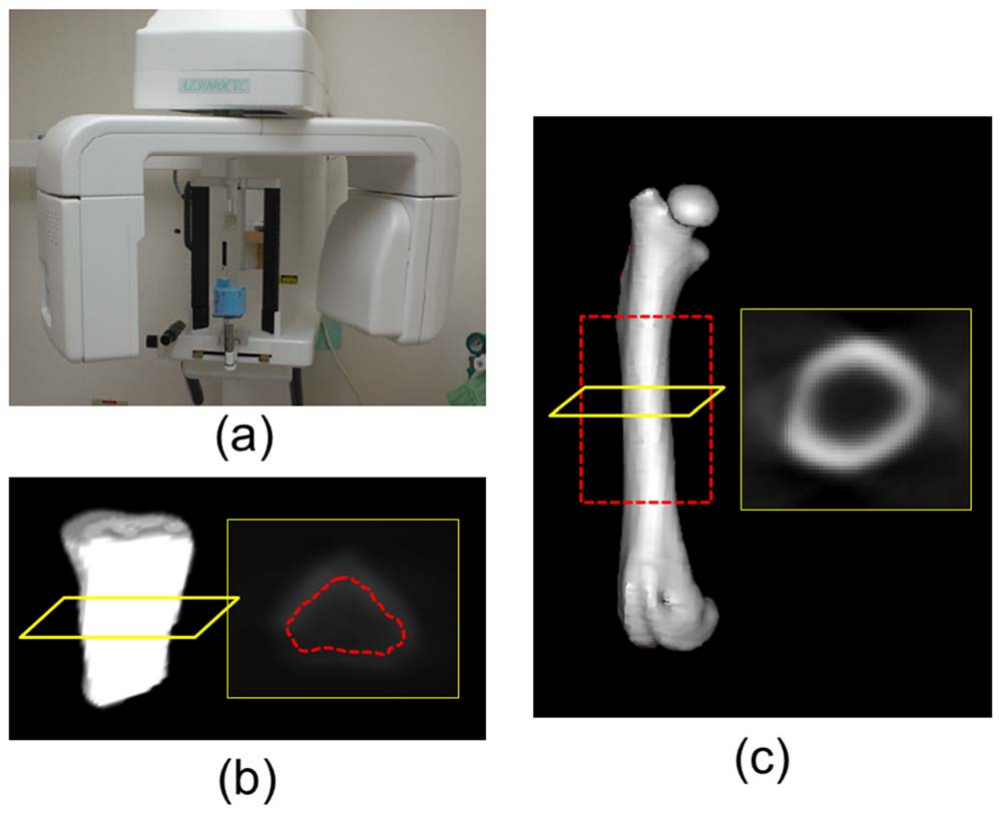
Dental CT machine, images and 3D renderings. (a) Dental CT machine (Asahi AZ 3000), (b) hemi 5th lumbar vertebral body (excluded the posterior element) in left, the section of the dental CT image in right (the region of interest for trabecular bone calculated also shown in dotted red line), and (c) intact femur (the region of interest for cortical bone calculated also shown in dotted red line) in left, the section of the dental CT image in right.

A dental CT device (AZ 3000, Asahi Roentgen, Japan) was used to obtain dental CT images of each femur (Fig. 2a). The scanning parameters were set at 85 kV, 3 mA, and a voxel resolution of 100 µm. In the dental CT approach, we used only one grayscale value (volumetric cancellous bone grayscale value, vCanGrayscale) to represent the cancellous bone of the fifth lumbar vertebral body because the resolution of dental CT is not sufficiently high for detecting the trabecular bone structure of rats. In addition, because identifying the border between the cortical and cancellous bone of a vertebral body is difficult, we first segmented the vertebral body (including the inner cancellous bone and outer cortical layer) and then eroded the segments using 3 voxel (0.3 mm) to exclude the cortical bone. Finally, the vCanGrayscale of the fifth lumbar vertebral body could be obtained (Fig. 2b). In addition, using a similar micro-CT approach, the TtAr, CtAr, CtAr/TtAr, and CtTh of the midshaft of the femurs (in the same region as that used for the micro-CT scans) were calculated (Fig. 2c). All measurements obtained in the dental CT approach were calculated using ImageJ.

### Statistical analysis

The mean, standard deviation, and coefficient of variation (CV) were calculated for all measurements. The Shapiro-Wilk test was used to determine if the measurements conformed to a normal distribution. The Pearson correlation coefficients (*r* values) between the vCanGrayscale measurements obtained using dental CT and the four trabecular bone microarchitecture parameters (BV/TV, TbN, TbTh, and TbSp) were calculated. Paired-sample *t* tests were used to compare the differences between the measurements of the four cortical bone morphology parameters (TbAr, CtAr, CtAr/TbAr, and CtTh) measured using micro-CT and the dental CT measurements. In addition, the Pearson correlation coefficients (*r* values) between these parameters measured using the two approaches were calculated. All statistical analyses of the data were performed using OriginPro software (version 8, OriginLab, Northampton, MA, USA). The level of statistical significance was set as *p*<0.05.

## Results

### Relation between the trabecular bone microarchitecture parameters measured using micro-CT and dental CT

**Figure 3:**
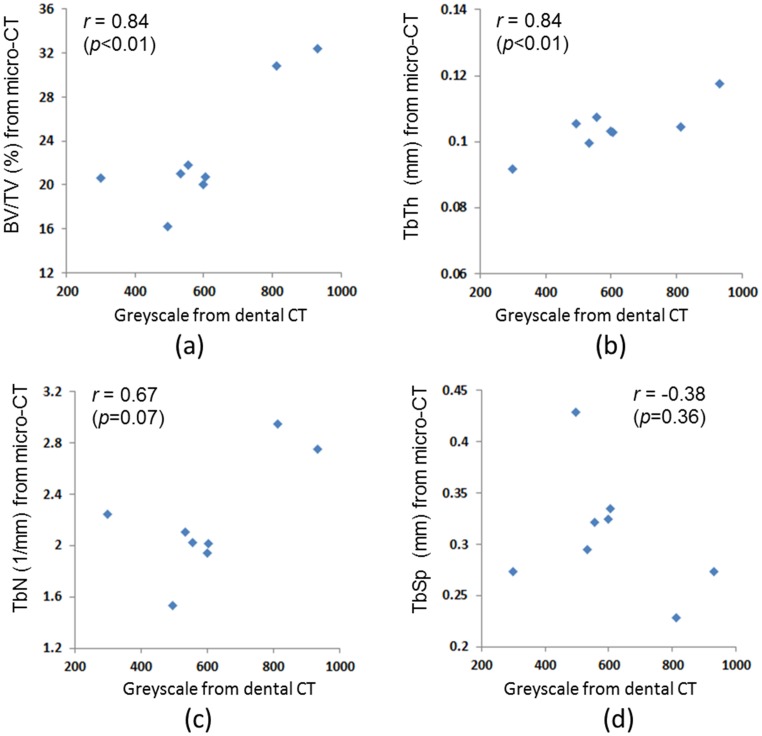
Correlations of the vCanGrayscale (grayscale value of the cancellous bone of 5^th^ vertebral body) measured using dental CT and micro-CT with BV/TV (a), (b) TbTh, (c) TbN, (d) TbSp.

The trabecular bone parameters of the fifth vertebral body measured using micro-CT and dental CT are listed in Table 2. All of the experimental data were normally distributed based on the Shapiro-Wilk test analysis. In the dental CT approach, the CV of the vCanGrayscale was 32.112%, which is higher than the CV of the four trabecular bone microarchitecture parameters (24.509%, 6.974%, 19.088%, and 20.769 for BV/TV, TbTh, TbSp, and TbN, respectively.) The correlation coefficient between the grayscale measured using dental CT and the BV/TV measured using micro-CT was 0.84 (*p*<0.01), which is slightly higher than the value of 0.84 (*p*<0.01), which was the correlation coefficient between the grayscale measured using dental CT and the TbTh measured using micro-CT (Fig. 3ab). These two coefficient values (both equal to 0.84) are all highly positive correlations. In addition, the correlation coefficients between the grayscale measured using dental CT and the TbN and TbSp measured using micro-CT were 0.67 (*p* = 0.07) and −0.38 (*p* = 0.36), respectively. However, both correlation coefficient values were nonsignificant (*p*>0.05) (Fig. 3cd).

**Table 2:** Trabecular bone parameters of the 5^th^ vertebral body: the four trabecular bone microarchitecture parameter (BV/TV, TbN, TbTh, TbSp) measured by micro-CT and the grayscale measured by dental CT.

Scanning type	Parameter	Unit	Mean±SD	CV (%)	Max	Min
Micro-CT	BV/TV	%	22.95±5.63	24.51	32.40	16.16
	TbTh	mm	0.10±0.01	6.974	0.12	0.09
	TbSp	mm	0.31±0.06	19.09	0.43	0.23
	TbN	1/mm	2.20±0.46	20.77	2.95	1.53
dental CT	vCanGrayscale		603.57±193.82	32.11	931.28	299.33

SD  =  standard deviation; CV  =  coefficient of variation (100×SD/mean); BV/TV  =  percent bone volume [bone volume (BV)/total volume (TV)]; TbTh  =  trabecular thickness; TbSp  =  trabecular separation; TbN  =  trabecular number; vCanGrayscale  =  volumetric cancellous bone grayscale value.

### Relation between the cortical bone morphology parameters measured using micro-CT and dental CT

**Figure 4:**
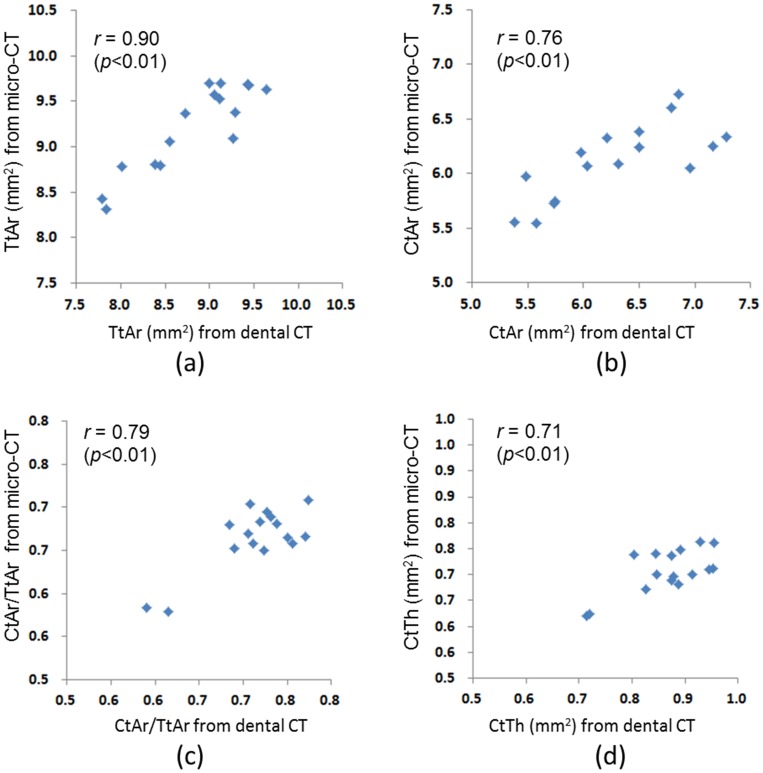
Correlations of the (a)TtAr, (b)CtAr, (c)CtAr/TtAr, (d)CtTh measured by micro-CT and dental CT.

The cortical bone morphology parameters of the femoral diaphysis measured using micro-CT and dental CT are listed in Table 3. All of the experimental data were normally distributed based on the Shapiro-Wilk test analysis. The TtAr parameter, which was measured using micro-CT (9.22±0.47 mm^2^), was significantly (*p*<0.01) larger than that measured using dental CT (8.82±0.59 mm^2^). However, the CtAr/TtAr and CtTh parameters, both measured using dental CT (0.71±0.05 for CtAr/TtAr, 0.87±0.07 for CtTh), were significantly (*p*<0.01) larger than that measured using micro-CT (0.66±0.04 for CtAr/TtAr, 0.71±0.03 for CtTh). For the CtAr parameter, no significant difference between the two approaches was observed (6.28±0.61 for dental CT and 6.11±0.34 for micro-CT). The correlation coefficient between the TtAr, CtAr, CtAr/TtAr, and CtTh measured using micro-CT and dental CT was 0.90 (*p*<0.01), 0.76 (*p*<0.01), 0.79 (*p*<0.01), and 0.71 (*p*<0.01), respectively (Fig. 4). All of these values indicated highly positive correlations.

**Table 3:** Cortical bone parameters of the femoral diaphysis: the four cortical bone morphology parameters (TbAr,CtAr, CtAr/TbAr, and CtTh) measured by micro-CT and dental CT.

	Parameter	Unit	Mean±SD	CV (%)	Max	Min
Micro-CT	TtAr	mm^2^	9.22±0.47	5.13	9.70	8.31
	CtAr	mm^2^	6.11±0.34	5.62	6.72	5.55
	CtAr/TtAr		0.66±0.04	5.51	0.71	0.58
	CtTh	mm	0.71±0.03	6.09	0.76	0.62
Dental CT	TtAr	mm^2^	8.82±0.59	6.70	9.64	7.79
	CtAr	mm^2^	6.28±0.61	9.74	7.29	5.38
	CtAr/TtAr		0.71±0.05	7.03	0.78	0.59
	CtTh	mm	0.87±0.07	8.42	0.96	0.71

SD  =  standard deviation; CV  =  coefficient of variation (100×SD/mean); TtAr  =  total cross-sectional area; CtAr  =  cortical area; CtAr/TtAr  =  cortical bone area fraction; CtTh  =  cortical thickness.

## Discussion

Measuring bone quality, quantity, and strength is a clinically crucial topic. Although micro-CT can be used to obtain trabecular bone microarchitectures and cortical bone morphology, this technology cannot be employed in measuring human bones because of the size limitations of such devices. Dental CT is becoming widely used in recent years. However, most previous studies on adopting dental CT to assess bone quality and bone quantity have been mainly concerned with presurgical dental implant assessments. According to our extensive research, no previous study has focused on the relationship between cortical bone morphology and trabecular bone microarchitecture by using both micro-CT and dental CT. The experiment conducted in this study revealed that, in measuring the four trabecular microarchitecture parameters of the cancellous bone of fifth lumbar vertebrae in rats, high correlations existed between the BV/TV [*r* = 0.84] and TbTh [*r* = 0.84] values measured using micro-CT and the vCanGrayscale values measured using dental CT. Similarly, the assessments of the four cortical bone morphology parameters of the femoral diaphysis in rats conducted using micro-CT and dental CT were highly correlated (*r* ranged from 0.71 to 0.90).

In laboratory experiments, the femoral diaphysis is one of the most frequently examined regions for measuring cortical bone strength because the region can be used to conduct three-point and four-point bending tests to measure the structural stiffness of the cortical bone [16], [25]. In addition, the femoral head and spinal vertebral body are generally selected to represent cancellous bone tissue [6], [8], [10], [11], [13]. This study adopted the fifth vertebral body of rats instead of the femoral head mainly because a rat's femur is small and contains insufficient cancellous bone. To prevent the image quality from being affected by the partial volume effects of dental CT, the fifth vertebral body of rats was used in this study as the sample of cancellous bone tissue. However, because the exterior of a vertebral body is covered by a thin cortical layer and the interior is covered by the cancellous bone tissue (Fig. 1b), dental CT with limited resolution cannot be used to accurately determine the trabecular bone microarchitecture in cancellous bone. Therefore, we first segmented the entire vertebral body, and then eroded the segments by 0.3 mm to represent the cancellous tissue inside the vertebral body.

Since Layton et al. [26] pioneered the use of micro-CT in analyzing the bone morphology of guinea pigs in 1988, micro-CT has been considered the gold standard for assessing bone morphology and microstructures [15]. Numerous bone parameters can be measured using micro-CT. In 2010, Bouxsein et al. [15] indicated that BV/TV, TbTh, TbSp, and TbN are the most crucial indices of trabecular bone microarchitecture parameters, and TtAr, CtAr, CtAr/TtAr, and CtTh are the most critical indices of cortical bone morphology parameters. Therefore, these eight parameters were adopted as the indices for assessing trabecular bone microarchitecture and cortical bone morphology parameters. According to a previous study, a rat's trabecular bone thickness and trabecular bone separation are approximately 50 µm and 150 µm, respectively [27]. The resolution of the micro-CT used in this study was 18.3 µm, which was sufficient for measuring trabecular bone microarchitectures. However, the resolution of the dental CT employed in this study was 100 µm, by which the trabecular bone microarchitectures could not be determined. Therefore, we adopted the vCanGrayscale to represent the cancellous bone tissue of the fifth vertebral body in rats.

Regarding the trabecular bone microarchitecture parameters of the cancellous bone, previous studies have used the femoral head of a rat as the typical region of interest [6], [10]. Because of the limited resolution of dental CT, the fifth lumbar vertebral body of rats was used in the experiment. In a comparison between this study and previous studies in which the third to fifth lumbar vertebral bodies of rats were measured, the BV/TV values (22.95%±5.63%) of the rats' fifth lumbar vertebral body scanned using micro-CT in this study were lower than those (29.18%±3.6%) measured by Ito et al. [8] or those (37.6%±5.0%) measured by Yao et al. [13]. This difference existed mainly because the 4-month-old rats used in this study were younger than the 10- and 6-month-old rats selected in the studies of Ito et al. [8] and Yao et al. [13], respectively. In addition, although the ratio of TbSp to TbTh in this study was fairly close to that reported in Ma et al. [11], the ratio of TbTh to TbSp in this study was greater than those obtained in previous research [8], [11], [13]. These value variations may have resulted from differences in the rat species used, rat age, and experimental design.

Regarding the cortical bone morphology parameters, micro-CT has been rarely applied to measure the four cortical bone morphology parameters (TtAr, CtAr, CtAr/TtAr, and CtTh) of a rat's femoral diaphysis. In this experiment, the TtAr and CtAr values of a the femoral diaphysis of rats measured using micro-CT were 9.22±0.47 mm^2^ and 6.11±0.34 mm^2^, respectively, which were only approximately half of the values (18.64±0.45 mm^2^ and 11.67±0.21 mm^2^, respectively) obtained by Sibilia et al. [28]. However, this study and the study of Sibilia et al. reported similar CtAr/TtAr values. In addition, the CtTh values (0.71±0.03 mm) obtained in this study were lower than those (1.095±0.03 mm) of Sibilia et al. In addition to the aforementioned differences in rat age, rat species, and experimental design, the partial volume effects of peripheral quantitative computed tomography (pQCT; resolution  =  70 µm) used by Sibilia et al. [28] may have caused measurement errors, which could have resulted in the differences in the absolute values.

In recent years, dental CT has been widely used in dental clinical practices mainly because dental CT is not only inexpensive and involves low radiation doses [18], [29], [30], but it also possesses higher spatial resolutions for precisely measuring bone shapes and contours than traditional computed tomography does. Hashimoto et al. [31] indicated that both the magnification and distortion of dental CT are extremely small (error < 0.1 mm). In addition to employing dental CT to observe tissue shapes and contours, several recent studies on the application of grayscale values measured using dental CT for determining bone density have demonstrated that bone quality and quantity have a specific relationship with grayscale values [22], [23]. However, because dental CT is generally used in dental clinics and by dentists or dental radiologists, most studies have been restricted to the dental field, which consequently reduced the applicability of dental CT to other orthopedic fields. Therefore, this study aimed to adopt dental CT to measure the cortical bone morphology parameters of a rat's femoral diaphysis and the grayscale values of the cancellous bone density of a rat's fifth vertebral body.

Previous studies have indicated that the image quality of dental CT is less stable than that of traditional computed tomography, and that the Hounsfield unit scale is not a suitable image unit in dental CT. Moreover, the image quality can be affected by the scanning position [19], [32]. Nevertheless, flat panel detectors have been used in most dental CT devices recently, which has substantially improved the image quality of dental CT [24]. Nomura et al. [33] also proved that the grayscale values measured using dental CT have a strong correlation with the concentrations of iodine solutions. Furthermore, Nomura et al. [24] demonstrated that dental CT can be employed to determine bone mineral content. In addition, dental CT has been adopted in numerous studies for assessing the bone quality and quantity of alveolar bone before dental implant placements. In this study, the vCanGrayscale values measured using dental CT represented the cancellous bone density of the fifth vertebral body, and exhibited high correlations (*r*) with the trabecular bone microarchitecture parameters, specifically BV/TV (0.84) and TbTh (0.84), measured using micro-CT. Therefore, dental CT can be clinically applied to scan patients' bones and indirectly estimate the trabecular bone microarchitecture (particularly the parameters of BV/TV and TbTh) by calculating the grayscale values of cancellous bone density. Additionally, although vCanGrayscale values were moderately correlated with TbN values (*r* = 0.67), the correlation exhibited no statistical significance (*p* = 0.070). Moreover, no correlations existed between the vCanGrayscale and TbSp values.

Several scholars have adopted QCT or pQCT to measure the morphology parameters of the femur [2], [3], [5], [34], and proved that QCT and pQCT can provide not only bone densitometric parameters as DXA does, but also bone geometric parameters for accurately predicting bone strength. However, scant studies have involved the use of dental CT for measuring the cortical bone morphology parameters of long bone diaphysis. In this experiment, the TtAr values (8.82±0.59 mm^2^) measured using dental CT were smaller than those (9.22±0.47 mm^2^) measured using micro-CT and, conversely, the CtAr/TtAr and CtTh values (0.71±0.05 mm and 0.87±0.07 mm, respectively) measured using dental CT were greater than those (0.66±0.04 mm and 0.71±0.01 mm, respectively) measured using micro-CT. Although differences existed in the absolute values measured using micro-CT and dental CT, the absolute values obtained using the two methods exhibited strong correlations. These absolute values should considerably decrease when the methods are applied in human clinical trials (thicker bones). However, further experiments are required to verify this assumption.

In this study, the experiment has several limitations. Because fresh human cadaver bones were difficult to obtain, the rat bones most commonly used in experiments were selected as the experimental specimens. Nevertheless, additional human bone experiments are required before these methods are applied to human bodies. In this study, only the femoral diaphysis was used to assess cortical bone, and cancellous bone was evaluated merely based on the fifth vertebral body. Consequently, the effectiveness of using other bone regions to test cortical and cancellous bone by using dental CT still requires further evaluation. In addition, all of the bone specimens were scanned in vitro by using micro-CT and dental CT, which generated clearer images than in vivo bone scanning did. Nonetheless, further experimental investigations are required for analyzing such differences.

## Conclusion

Based on the experimental setup and limitations, the following conclusions were derived from this study:

1. High correlations between the vCanGrayscale (grayscale value of the cancellous bone of the fifth vertebral body) measured using dental CT and the trabecular bone microarchitecture parameters (BV/TV and TbTh) measured using micro-CT were observed.
2. The absolute value of the cortical bone morphology parameters (TtAr, CtAr/TtAr, and CtTh) may be different between the measurements obtained using the dental CT and micro-CT approaches. However, high correlations between these four parameter measured using micro-CT and dental CT were demonstrated.
